# Radiofrequency Echographic Multispectrometry (REMS): A New Option in the Assessment Bone Status in Adults with Osteogenesis Imperfecta

**DOI:** 10.3390/jimaging9100210

**Published:** 2023-10-03

**Authors:** Carla Caffarelli, Antonella Al Refaie, Caterina Mondillo, Alessandro Versienti, Leonardo Baldassini, Michela De Vita, Maria Dea Tomai Pitinca, Stefano Gonnelli

**Affiliations:** Division of Internal Medicine, Department of Medicine, Surgery and Neuroscience, University of Siena, 53100 Siena, Italy; antonellaalrefaie@gmail.com (A.A.R.); caterinamondillo28@gmail.com (C.M.); leo.balda90@libero.it (L.B.); dea_to79@yahoo.it (M.D.T.P.); gonnelli@unisi.it (S.G.)

**Keywords:** osteogenesis imperfecta, bone mineral density (BMD), dual-energy X-ray absorptiometry (DXA), radiofrequency echographic multispectrometry (REMS), fragility fractures

## Abstract

This study aimed to estimate the utility of the Radiofrequency Echographic Multispectrometry (REMS) approach in the assessment of bone mineral density (BMD) in subjects with osteogenesis imperfecta (OI). In 41 subjects (40.5 ± 18.7 years) with OI and in 36 healthy controls, we measured BMD at the lumbar spine (LS-BMD), femoral neck (FN-BMD) and total hip (TH-BMD), employing a dual-energy X-ray absorptiometry tool. Additionally, REMS scans were also performed at the lumbar and femoral sites. The presence and number of reported fractures were assessed in the study population. Patients characterized by a history of fragility fractures represented 84.5% of the study population. OI subjects showed significantly reduced BMD values both at the level of the lumbar spine and the femoral subregions (*p* < 0.01) compared to healthy controls when performed using both the DXA and the REMS method. Dividing OI patients on the basis of the Sillence classification, no differences were found between the LS-BMD values carried out using the DXA technique between the OI type I group and OI Type III and IV groups. On the contrary, the OI Type III and IV groups presented significantly lower values of both Trabecular Bone Score (TBS) and LS-BMD through REMS with respect to OI type I patients (*p* < 0.05). Based on the data of this study, it is possible to conclude that even the new REMS assessment, which does not use ionizing radiation, represents an excellent method for studying the bone status in subjects affected by OI.

## 1. Introduction

Osteogenesis imperfecta (OI) is a rare (affecting one in 15–20,000 individuals) hereditary disorder of connective tissue, mainly characterized by qualitative and quantitative alterations of bone collagen responsible for bone fragility and increased risk of fractures [1,2]. Until recently, only the mutations in one of the two genes coding for collagen type I alpha chains (COL1A1/COL1A2) were associated with OI, the so-called “classical mutations”, whereas, in recent years, other mutations (“non classical mutations”) have been discovered [1,2]. They encode genes involved in the folding or in post-translational modification processes of collagen, in osteoblast differentiation and in bone mineralization [1]. This genetic heterogeneity also corresponds to a clinical heterogeneity. The most common clinical features in OI are frequent fractures that are often precipitated in the absence of trauma or by minimal trauma, blue sclera, dentinogenesis imperfecta, joint hyperlaxity, short stature, progressive bone deformities, hearing impairment and muscle weakness. In 1979, in a previous paper by Sillence et al., a classification of OI based on clinical severity and radiographic criteria was proposed, which is still used [3]. According to the Sillence classification, OI type I is the mildest clinical form and is characterized by a mainly quantitative reduction in type I collagen; OI type III is the most severe non-lethal form, while OI type IV has an intermediate phenotype between types I and III. OI type II is not found in adults, because it is lethal in the perinatal period [3]. 

The patients with OI have an increased lifetime risk of fractures compared with the healthy general population. In particular, the fracture rate peaks during childhood and adolescence and then decreases during adulthood and increases again in women after menopause [4,5]. Many studies have reported that in OI, bone microarchitecture is markedly altered. In fact, trabecular thickness and volumetric bone mass are lower; cortical thickness is also reduced with higher intracortical porosity [6,7]. All these alterations in bone structure alter the bone biomechanics and increase the bone fragility of subjects with OI [7,8]. Regarding markers of bone turnover, all previous studies have detected normal or slightly increased values of both bone formations compared to the bone resorption markers [9,10]. Bone mineral density (BMD) through dual-energy X-ray absorptiometry (DXA) is undoubtedly useful for assessing disease progression and the response to bisphosphonate treatments in patients with OI. However, numerous studies have demonstrated that in many patients with OI, BMD through DXA is only slightly reduced and sometimes even increased [11,12]. Furthermore, the measurement of lumbar spine BMD is often made problematic by the presence of scoliosis and previous fractures [11,12]. These data highlight that DXA is not always able to reflect bone quality and bone fragility in OI individuals. High-resolution–peripheral quantitative computed tomography (HR-pQCT) better characterizes bone microarchitecture and is able to measure cortical and trabecular microstructural compartments. However, due to its technical characteristics and cost, HR-pQCT cannot be considered a diagnostic tool for routine use [11]. Therefore, there is currently a growing interest in having reliable and easy-to-use diagnostic tools to evaluate bone quality in OI patients. Trabecular bone score (TBS) has shown a good correlation with bone microstructure parameters obtained using HR-pQCT and could provide information on some parameters of bone quality in OI [11,13]. 

A more recent and attractive tool is Radiofrequency Echographic Multispectrometry (REMS) technology. REMS is founded on the testing of native raw unfiltered radiofrequency (RF) ultrasound signals, obtained while performing an echographic exam of proximal femur and lumbar vertebrae [14]. The assessment of unfiltered native ultrasound signals allows the capture of more information on the features of the bone tissues evaluated, which are normally filtered during the conventional B-mode image rebuilding process [14]. Several recent studies have shown a good diagnostic agreement between the BMD values obtained using the REMS technique compared to the DXA method. Moreover, REMS assessment was characterized by good repeatability and adequate precision but it was also able to predict the risk of fragility fractures [15,16,17]. In addition, REMS technology seems to be able to overcome some limitations of DXA, such as the overestimation of BMD in the presence of artifacts, osteophytes and fractures, and also to provide bone qualitative information [18,19]. 

This research aimed to assess the clinical utility of REMS technology in the evaluation of bone mineral density in adult patients with osteogenesis imperfecta. 

## 2. Materials and Methods

We conducted an observational retrospective case–control study at the outpatient Clinic for Osteoporosis of the Department of Internal Medicine at the University Hospital of Siena (Italy) between January 2022 and December 2022.

### 2.1. Study Population

We studied 41 patients (21 males and 20 females) with osteogenesis imperfecta who were followed in the outpatient Clinic for Osteoporosis of the Department of Internal Medicine, Surgery and Neuroscience at the University Hospital of Siena (Italy). Subjects with clinical or genetic diagnosis of OI type I, III or IV were enrolled in this study. Similarly, subjects with all secondary causes of osteoporosis were excluded from inclusion in this study. For each patient with OI, we collected a detailed clinical history especially focused on fractures (intrauterine fractures, time of the first fracture, fracture rate and location, etc.). We classified patients with osteogenesis imperfecta according to the Sillence classification. In particular, they were grouped according to genetic diagnosis and phenotypic expression of the disease [3]. Therefore, OI subjects were divided into OI Type I (*n* = 32, 78.0%), OI Type III (*n* = 5; 12.2%) and OI Type IV groups (*n* = 4; 9.8%). All OI patients were taking oral supplementation with calcium (500–1000 mg daily) and cholecalciferol (800 IU daily). Twenty-five patients (63%) were on neridronate treatment. This protocol provides for the administration of neridronate (2 mg/kg, up to a maximum of 100 mg) every 3 months. The control group included 36 healthy age-matched subjects. In all subjects, the following anthropometric variables were tested in standard conditions: weight, stature and body mass index. The body mass index (BMI) expresses the ratio of weight in kilograms divided by height in meters squared. All participants gave informed consent and this study was performed according to the Declaration of Helsinki and was approved by the Institutional Review Board of Siena University Hospital (ID-1234/14). 

### 2.2. Biochemical Parameters

In our study population, fasting venous blood samples were collected in order to measure serum levels of creatinine, alkaline phosphatase (ALP), calcium and phosphate. Moreover, we evaluated bone turnover markers, particularly type I collagen β carboxy telopeptide (βCTX) and bone alkaline phosphatase (B-ALP). In our institution, an enzyme-linked immunoassay method (Immunodiagnostic Systems, Boldon, UK) was used for the determination of serum βCTX; in this case, the intra- and inter-assay coefficients of variation were 2.5% and 4.0%, respectively. The serum B-ALP was evaluated using a chemiluminescence immunoassay method (LIAISON BAP Ostase, DiaSorin Inc., Stillwater, MN, USA). In our laboratory, the B-ALP the intra-and inter-assay coefficients of variation were 4.2% and 7.9%, respectively. We also measured the following hormones in all subjects: (1) Parathyroid hormone (PTH), which was evaluated using an immunoradiometric assay (Total Intact PTH, Antibodies Lab. Inc.; Santee, CA, USA). In our laboratory, the intra- and inter-assay coefficients of variation were 3.6% and 4.9%, respectively. (2) 25-hydroxyvitamin D (25OHD), which was measured using a chemiluminescence immunoassay (LIAISON 25OHD Total Assay, DiaSorin Inc., Stillwater, MN, USA). In our laboratory, the inter- and intra-assay coefficients of variation were 9.2% and 6.8%, respectively. 

### 2.3. Bone Mineral Density Measurement Using DXA

We evaluated bone mineral density using DXA both in subjects affected by osteogenesis imperfecta and in controls. In particular, we measured the BMD at the level of the first 4 vertebrae of the lumbar spine (LS-BMD) and at the level of the proximal femur taking into account the region of the femoral neck (FN-BMD) and the total hip (TH-BMD). All BMD scans were made using dual-energy X-ray absorptiometry (Discovery W, Hologic, Waltham, MA, USA). 

In all patients with osteogenesis imperfecta and in controls, DXA evaluations were performed according to the standardized clinical routine procedures. Moreover, the diagnosis of osteoporosis and osteopenia was carried out based on the definition by the World Health Organization (WHO) and according to ISCD guidelines [20]. In particular, osteoporosis is defined by a T-score of −2.5 standard deviations (SDs) or less, osteopenia is defined by a T-score between −1.0 and −2.5 SDs, and normal values of BMD are determined by a T-score higher than −1.0 SD. For the testing of Z-score and T-score, sex-matched Italian reference data were used. For a better estimate of bone tissue microarchitecture, we also calculated the Trabecular Bone Score (TBS). TBS was calculated by using TBS iNsight software (Version 2.1, Medimaps SA, Bordeaux, France) in an operator-independent automated manner. The Trabecular Bone Score was calculated from the standard DXA scan of the antero-posterior lumbar spine. TBS calibration phantom (25% fat mass equivalent and 17 cm thickness) was employed for TBS value calibration and were adjusted for BMIs up to 21.78. The short-term precision of TBS assessment was 1.5% (CV) in our outdoor clinic for densitometry test.

### 2.4. Bone Mineral Density Measurement Using REMS 

**Figure 1:**
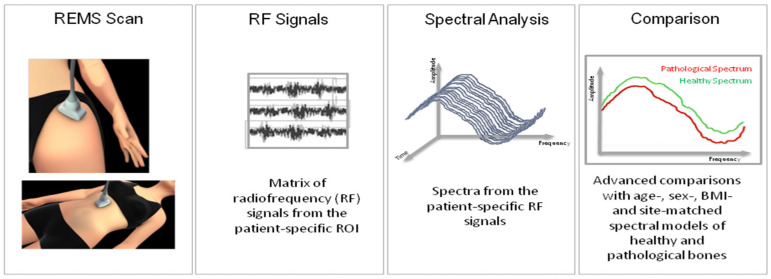
Schematic explanation of Radiofrequency Echographic Multispectrometry (REMS) assessment on lumbar spine and femoral site.

We conducted a bone mineral density assessment at the axial site level for all participants in this study using the REMS technique. The REMS assessment was carried out at both lumbar spine and femoral sites by using a dedicated echographic device (EchoStation, Echolight Spa, Lecce, Italy), provided with a convex transducer functioning at a nominal frequency of 3.5 MHz. During an evaluation using the REMS method with the patient lying on their back, the ultrasound probe was positioned on the femur or on the abdomen, in order to identify the site of interest (femoral neck or lumbar spine). The operator would have to set the appropriate value of depth and the correct focus of the transducer. In particular, once an ultrasound scan has been carried out on the lumbar vertebrae or proximal femur, the software allows the detection, automatically, of the target bone structure and the identification of the Region of Interest (ROI) in the trabecular part of the bone structure. Subsequently, in the identified ROI, the software will analyze, in a completely automatic and operator-independent manner, the spectral characteristics of the raw ultrasonic signals (RF). In particular, the software performs a series of dedicated statistical and spectral processing, on the corresponding ultrasonic “raw” signals, whose spectra are compared analytically with a database of “model spectra” (healthy bone models and osteoporotic) specific for age, BMI, corresponding gender and ethnicity to those of the patient under examination. Precisely, the spectral changes provided by the physical characteristics of the skeletal structure that has backscattered the ultrasound are revealed by the comparison process, resulting in a BMD evaluation. At the end of this analysis, the software outputs the parameters BMD, T-Score and Z-score and allows the categorization of osteoporosis, osteopenia and normal BMD [14,18,21]. Figure 1 shows a schematic representation of the REMS acquisition at the lumbar spine and proximal femur.

### 2.5. Statistical Analysis 

All values reported are expressed as mean ± standard deviation (SD). Mann–Whitney U-test or Student’s *t* test was employed to compare the variables considered in the above study groups. The Kolmogorov–Smirnov test was used to verify normality distribution. Spearman’s correlation coefficient was used in order to evaluate correlation degree between TBS, DXA and REMS values. All statistical analysis were carried out using the SPSS statistical package for Windows version 16.0 (SPSS Inc., Chicago, IL, USA).

## 3. Results

Demographic, clinical and densitometric parameters of the study populations are shown in Table 1. Patients with OI had a mean age of 40.5 ± 18.7 years and were therefore similar to that of the control group. As expected, weight and height were significantly lower in OI patients with respect to the healthy control group (*p* < 0.01); on the contrary, 25OHD serum levels were significantly increased in OI subjects than in controls. This is because all OI patients were taking vitamin D supplements. Bone mineral density evaluated using DXA and REMS methods at all skeletal regions (TH-BMD, FN-BMD and LS-BMD) and TBS were all significantly (*p* < 0.01) reduced in patients with osteogenesis imperfecta with respect to the healthy control subjects (Table 1). 

**Figure 2:**
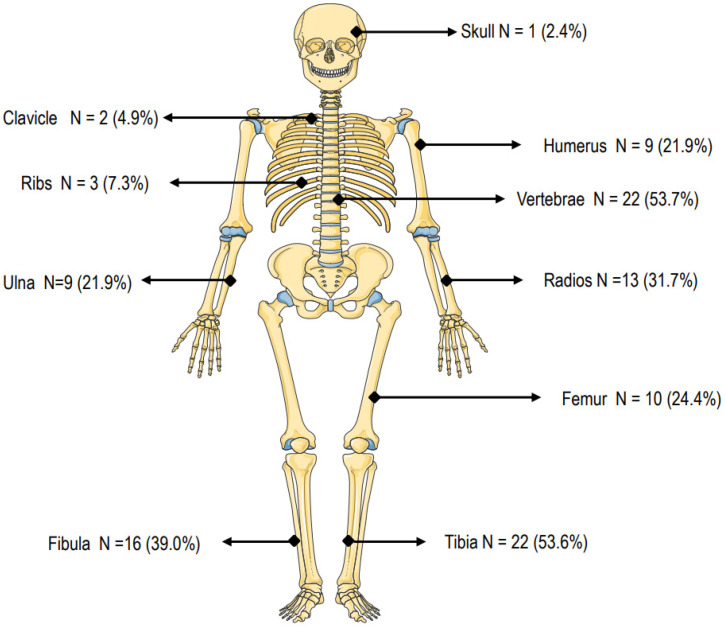
Number and location of fragility fractures in a cohort of 41 adult patients with osteogenesis imperfecta.

Moreover, 35 (85.4%) OI patients had experience of fractures. In particular, 22 OI patients had a history of vertebral fractures and 10 OI patients reported hip fractures, whereas tibia or fibula fractures were present in 38 subjects. As expected, all patients with OI Type III and IV had a history of multiple fractures. In addition, two OI patients had fractures of the skull and femur at birth. The distribution of fractures at various skeletal sites is detailed in Figure 2. 

**Figure 3:**
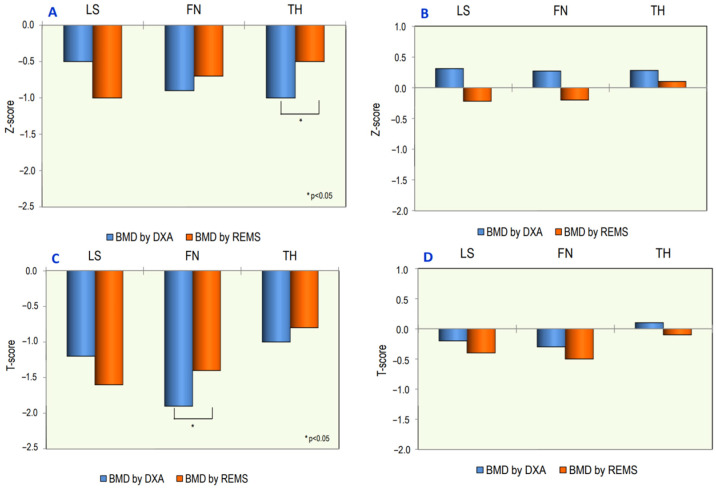
Values of BMD expressed as Z-score and T-score at lumbar spine (LS), at femoral neck (FN) and at total hip (TH) using DXA and REMS techniques in patients with osteogenesis patients (**A**,**C**) and healthy controls (**B**,**D**).

The mean values of BMD using DXA and REMS, at different skeletal sites, in OI patients and in healthy controls, expressed as Z-score and T-score, are shown in Figure 3. TH-BMD and FN-BMD Z-score values using REMS were higher with respect to those using DXA but reached statistical significance only at TH-BMD (*p* < 0.05). On the contrary, at the lumbar spine, the BMD Z-score using REMS was lower with respect to BMD using DXA. In addition, the BMD values expressed as T-score had the same course; in this case, statistical significance was reached only at the FN-BMD T-score. No significant differences in T-score and Z-score using DXA and REMS techniques were observed in the control group (Figure 3).

**Figure 4:**
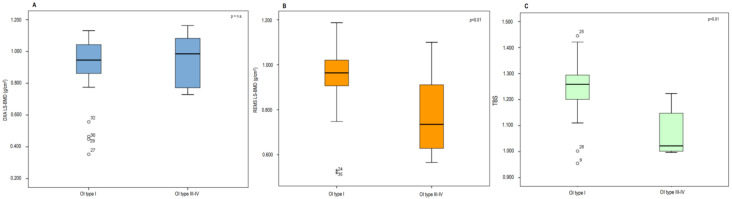
LS-BMD through DXA (**A**), LS-BMD through REMS (**B**) and TBS (**C**) in subjects with OI type I and OI type III–IV. n.s. = not significant.

The values of LS-BMD, using both DXA and REMS techniques, and those of TBS assessed in subjects with OI type I and OI Type III and IV, respectively, are shown in Figure 4. There were no differences between the LS-BMD values carried out using the DXA technique between the OI type I group and OI Type III and IV groups. On the contrary, the OI Type III and IV groups showed significantly lower values of both TBS and LS-BMD using REMS with respect to patients suffering from OI type I (*p* < 0.05). Moreover, a significant correlation was found between BMD using DXA and BMD using REMS at both the femoral neck (r = 0.54, *p* < 0.01) and total hip (r = 0.65, *p* < 0.01) in subjects suffering from OI. In these patients, a significant but lower correlation was also discovered at the level of the lumbar spine (r = 0.35, *p* < 0.05).

**Figure 5:**
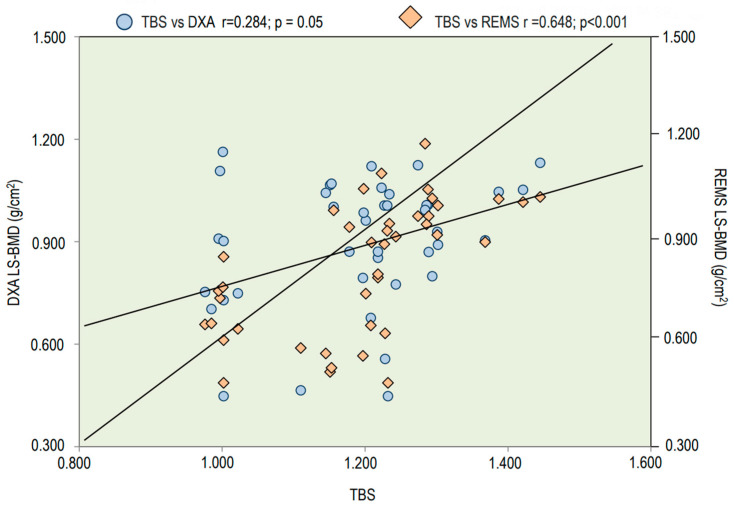
Spearman’s correlation of TBS and LS-BMD through DXA and REMS techniques.

Figure 5 reports Spearman’s correlation of TBS values with LS-BMD using both DXA and REMS techniques. The TBS values presented significant correlations with both LS-BMD through the DXA technique and LS-BMD through the REMS technique; however, a better correlation was observed between TBS values and LS-BMD through REMS (r = 0.648; *p* < 0.001).

## 4. Discussion

This is the first study that has considered the usefulness of new REMS technology in adult patients with osteogenesis imperfecta. The data obtained from this preliminary study have shown that there is a good correlation between the values of BMD obtained through DXA and the values of BMD obtained through REMS at all skeletal sites. Therefore, based on this result, it would be possible to evaluate bone status in OI through REMS without using ionizing radiation. This finding is relevant, because OI patients, given their high fracture risk, require frequent radiological examinations for suspected fractures. Furthermore, they also demand periodic monitoring of bone mineral density assessment and radiological evaluation for vertebral fractures and spinal deformities with a consequent increase in cumulative radiation exposure. Moreover, some studies have shown that, in OI patients, the cumulative radiation dose could represent an additional lifetime cancer risk [22]. Therefore, the use of a technique free of ionizing radiation, such as REMS, could be particularly advantageous during adolescence and in women of childbearing age or during pregnancy and breastfeeding [23,24]. The ease of execution and the portability of REMS allow its use even at the bedside and in OI patients with many fractures and bone deformities, in whom DXA is not easily feasible, due to technical limitations.

Another interesting result of this study was the finding of a significant association between REMS and TBS. The literature data indicate that in many patients with OI, BMD through DXA is only slightly reduced and sometimes even increased; moreover, only a small percentage of patients display osteoporotic T-scores [25]. Therefore, the high risk of fragility fractures of OI patients cannot be fully explained by low BMD.

In fact, in OI patients, skeletal fragility is associated with reduced bone quality due to defects in bone mineralization and the bone matrix, which add to the alteration in bone microarchitecture. In particular, the reduction in cortical thickness and the higher cortical porosity alter bone biomechanics and reduce mechanical strength [6,7,8]. TBS is a gray-level measurement, derived from DXA images, which is known to provide information on trabecular microarchitecture, as assessed using central and peripheral QCT, and to predict fragility fractures in osteoporotic women and elderly men independently from BMD [26,27,28]. In our study, the good correlation between LS-BMD through REMS and TBS would confirm how the REMS method could be used to evaluate other bone qualities over BMD. This hypothesis is confirmed by the fact that, in our study, we observed that the values of both TBS and LS-BMD through REMS were significantly lower in OI-III–IV than in OI-I patients, while LS-BMD through DXA did not present significant differences between the two groups. This finding is in agreement with previous studies that reported that TBS is better than BMD through DXA in discriminating between patients with moderate to severe OI-III–IV and those with the mildest OI-I and may be explained by the fact that scoliosis and previous fractures, present in about 50% of adult OI patients, represent a limitation for LS-BMD through DXA [11,29]. Moreover, Hald et al. reported that patients with OI type III have reduced values of aBMD and vBMD (assessed through HR-pQCT) compared to patients with less severe OI [30]. Several recent studies have reported how fragility fracture represents an important problem in patients with OI even during adulthood [25,31]. Particularly, in adults with OI, the risk of fragility fractures in addition to reduced bone strength also seems to be increased through altered biomechanical muscle-bone relationships [31]. 

To date, only few studies have examined the number and location of fractures in adult patients with OI. Wekre et al. reported that most of the fractures were at the level of long bones of the lower and upper limbs or small bones of the hands and feet, and only 30% of patients reported vertebral fractures, but more than half of patients had never undergone spinal X-ray examination [25]. Instead, in our study, vertebral fractures were detected in over 50% of OI patients. This finding appears to be in agreement with literature data, reporting a high number of vertebral fractures in children with OI [32].

In particular, Amor et al. observed vertebral fractures in 71% of pediatric patients who had spine radiographs [32]. Vertebral fractures represent the most significant complications in OI patients because they lead to cardiopulmonary morbidity and thus to a lower quality of life. Therefore, the need to predict these fractures is fundamental. In agreement with what has been reported by other studies, we observed that most vertebral fractures were located in the lower thoracic tract [32]. Moreover, the presence of severe or moderate vertebral fractures, mostly localized in the thoracic tract, can cause a worsening in lung capacity, contributing to a worsening in the restrictive ventilator defect [33].

The high correlation we observed between REMS and TBS in our study suggests that REMS could become an important tool in the follow-up of adult patients with OI, able to assess the risk of fragility fractures and thus helping to overcome the limitations of DXA. It would also be interesting to understand the role of REMS in this delicate subgroup of patients. Furthermore, due to its technical characteristics, REMS could be an important diagnostic tool for the follow-up of patients, especially with regard to diagnostic and therapeutic monitoring. 

The limitations of this study are as follows: firstly, the cross-sectional nature of this study does not allow the establishment of any causality relationships between parameters; secondly, there was a small number of OI patients; thirdly, there was a lack of evaluation using HR-pQCT. 

This study presents some strong points too. Firstly, a healthy control group that was comparable in age and sex was available, and secondly, this was a single-center study and all densitometric tests through DXA and REMS were carried out by the same operator with adequate experience. 

This study pointed out that REMS could find interesting uses in people with rare skeletal diseases with increased fracture risk. In addition, the transportability of the device allows its use both at the patient bedside and in other clinical settings. Moreover, the fact that REMS is a technique free of ionizing radiation allows its safe use in the assessment of bone status in young people and especially in women during pregnancy and lactation. Based on these innovative features of REMS technology, this study may be a starting point for future research on the potential role of the REMS technique in bone evaluation and in longitudinal monitoring throughout life.

## 5. Conclusions

The findings of this study indicate that the longitudinal assessment of bone mineral density and of fracture risk using the REMS technology represents a new, useful and promising tool in patients with osteogenesis imperfecta. Moreover, REMS technology, similarly to TBS, can identify severe bone status impairment between patients with OI-I and OI-III–IV. Further investigations are necessary to confirm these preliminary results, but also, above all, to find out new parameters derived from REMS analyses that best identify bone quality. This would be especially important to improve the identification of fracture risk in OI patients. 

## Figures and Tables

**Table 1:** Characteristics of the study group.

	Osteogenesis Imperfecta (N = 41)	Controls (N = 36)	*p*
Age (years)	40.5 ± 18.7	41.7 ± 16.3	n.s.
Weight (kg)	61.2 ± 16.2	71.5 ± 12.2	0.01
Height (cm)	152.6 ± 19.5	169.5 ± 7.6	0.01
BMI (kg/m^2^)	26.1 ± 6.9	24.7 ± 2.9	n.s.
Creatinine (mg/dL)	0.9 ± 0.3	0.9 ± 0.2	n.s.
Calcium (mg/dL)	9.4 ± 0.4	9.2 ± 0.5	n.s.
Phosphate (mg/dL)	3.3 ± 0.6	3.4 ± 0.5	n.s.
25OHD (ng/mL)	38.0 ± 9.9	24.4 ± 8.9	0.05
PTH (pg/mL)	34.3 ± 19.7	36.8 ± 17.9	n.s.
B-ALP (µg/L)	13.2 ± 6.2	15.2 ± 8.9	n.s.
β-CTX (ng/mL)	0.377 ± 0.180	0.405 ± 0.142	n.s.
DXA LS-BMD (g/cm^2^)	0.906 ± 0.201	1.083 ± 0.126	0.001
DXA FN-BMD (g/cm^2^)	0.724 ± 0.168	0.886 ± 0.126	0.001
DXA TH-BMD (g/cm^2^)	0.789 ± 0.206	0.989 ± 0.112	0.001
TBS	1.209 ± 0.127	1.412 ± 0.100	0.001
REMS LS-BMD (g/cm^2^)	0.885 ± 0.105	1.000 ± 0.072	0.003
REMS FN-BMD (g/cm^2^)	0.714 ± 0.120	0.810 ± 0.093	0.001
REMS TH-BMD (g/cm^2^)	0.880 ± 0.132	0.955 ± 0.103	0.01

n.s. = not significant.

## Data Availability

Not applicable.
